# Evaluation of KO-Tab 1-2-3^®^: a wash-resistant 'dip-it-yourself' insecticide formulation for long-lasting treatment of mosquito nets

**DOI:** 10.1186/1475-2875-4-52

**Published:** 2005-11-03

**Authors:** Alison Yates, Raphael N'Guessan, Harparkash Kaur, Martin Akogbéto, Mark Rowland

**Affiliations:** 1 London School of Hygiene & Tropical Medicine, London, WC1E 7HT, UK; 2 Centre de Recherche Entomologique, Cotonou, Benin

## Abstract

**Introduction:**

Insecticide-treated nets (ITN) are an important method of preventing malaria. To remain effective, they need to be re-treated with pyrethroid insecticide at approximately yearly intervals. Systems for re-treating nets in Africa are limited, and the vast majority of nets in use have never been treated or were treated only once. Bayer Environmental Science (BES) has developed a long-lasting formulation 'KO-Tab 1-2-3^®^' which can be applied to the net post-manufacture, under field conditions, and renders the insecticide wash-resistant.

**Methods:**

The performance of polyester nets treated with three kinds of BES long-lasting formulations, a conventional ITN (treated with standard KO-Tab) and PermaNet 2.0 were evaluated after washing samples of treated netting up to 30 times using standard WHO procedures. Performance was measured using 'three-minute exposure' and 'median time to knockdown' bioassay tests and by measuring the levels of deltamethrin using high-pressure liquid chromatography.

**Results:**

The conventional ITN was largely stripped of deltamethrin within 5–10 washes and insecticidal efficacy in bioassay declined to suboptimal levels. With PermaNet and KO-Tab 1-2-3 the loss of deltamethrin was much slower: insecticide content halved within 20 washes and there was no loss of biological efficacy in three-minute exposure bioassays in WHO cylinders even after 30 washes. After 30 washes there remained on the netting 16% (4.4 mg/m^2^) of the loading dose of KO-Tab 1-2-3 and 28% (18.8 mg/m^2^) of the loading dose of PermaNet.

**Conclusion:**

KO-Tab 1-2-3 was confirmed to be a long-lasting insecticide formulation. This finding raises the prospect of conventional polyester nets being converted into long-lasting insecticidal nets through simple dipping in the community or at home. This single development, if widely adopted, could transform the malaria control landscape in Africa and have a major impact on malaria.

## Introduction

Insecticide-treated nets (ITN) are an effective malaria control tool but to remain effective need to be retreated with pyrethroid insecticide about once a year [1]. This is a major constraint in developing countries where the infrastructure to provide repeated treatment of nets is inadequate or absent. The advent of long-lasting insecticidal nets (LLIN) has provided a technical solution to this problem [2-7]. With this technology, surface insecticide is replenished either from a resin matrix that coats the surface of fibres or by diffusion through the fibres as outer insecticide is removed during washing and normal use [8-10]. International donors and institutional buyers are increasingly opting for LLIN as their preferred choice of net. However, the majority of ITN in current use, particularly those available through the commercial retail sector, are not LLIN. Indeed the majority of nets in current use have either never been treated [11] or were treated only on purchase or distribution, and when non-insecticidal nets develop holes, which they inevitably do, they offer little or no protection against malaria [12].

Current LLIN are treated with insecticide during net manufacture. Bayer Environmental Sciences (BES) has developed a formulation technology 'KO-Tab 1-2-3' which offers the prospect of conventional nets being converted into LLIN through a dipping process that can be done post-manufacture under field conditions or in the home. A dip-it-yourself long-lasting treatment could solve the problem of having to regularly retreat conventional ITN.

This report describes the evaluation of polyester nets treated with 3 types of long-lasting formulation produced by BES, also a commercially available LLIN 'PermaNet 2.0^®^' and a conventionally treated net. Nets were washed up to 30 times and evaluated using two types of WHO bioassay test and by chemical residue assay using high-pressure liquid chromatography assay of residual deltamethrin.

## Materials and methods

### Preparation and washing procedure

Three treated nets were prepared by BES: KO-Tab 1-2-3 in which a conventional KO Tab (suspension concentrate formulation) is mixed in aqueous solution with a special binder to make a long-lasting wash-resistant treatment; BES902 in which a conventional KO-Tab is mixed with a different type of binder; and BES903 which has double the quantity of deltamethrin and binder. A long-lasting insecticidal net (PermaNet^®^) served as a positive control. One conventional KO-Tab treated net was prepared at LSHTM for comparison. The nets were laid flat for drying in a room kept at 25°C, and turned at 10 minutes intervals.

Fifteen samples of netting, each measuring 120 × 30 cm, were cut from each net. The samples were kept in triplets (one sample from each side and top of the net) and washed 0, 5, 10, 20 or 30 times. This scheme was expected to adjust for any variation in deposition due to uneven treatment or drying.

The netting was washed according to standard WHO procedures [10] in 2 g/litre soap solution (Le Chat Paillettes^® ^Savon de Marseille) in deionised water at pH10-11. Net samples were washed individually for 10 minutes in 1 litre bottles containing 750 ml soap solution kept at 30°C in a motorized water bath adjusted to make 155 back-and-forth movements per minutes. The samples were then rinsed twice with clean deionised water at 30°C using the same procedure. The net samples were hung to dry at 24°C. There was a 24 h interval between washings and the 30 washings were carried out over six weeks.

Each 120 × 30 cm net sample was then sectioned into 3 pieces. The first piece was used for three minutes exposure bioassays, the second for median time to knock down tests, and the third for chemical assay.

### Median time to knock down bioassay tests

Median time to knock down (MTKD) bioassays were performed at LSHTM using the wire ball frame technique [13]. In MTKD bioassays 11 female *Anopheles stephensi *(BEECH, insecticide susceptible strain), 2–5 days old, were exposed to test netting in a metal frame and the time taken for the median (6^th^) mosquito to be knocked down was recorded. Knockdown was defined as collapsed against the netting or fallen to the base of the ball frame and not moving. As each mosquito was knocked down it was removed using a mouth aspirator so if it did recover it would not be counted again. The time for the median mosquito to be knocked down was the end point of the test. Three replicate tests were carried out on each piece of net, giving a total of 9 replicates for each treatment/wash combination.

### Three-minute exposure bioassay tests

Three-minute exposure bioassays were carried out by LSHTM staff based at the Centre de Recherche Entomologique de Cotonou in Benin. This type of test can be done using WHO plastic cones, wire ball frames, or WHO susceptibility test kits [13]. WHO cones are rather awkward and time-consuming for conducting tests of three minutes duration and present a large, non-insecticidal surface area if the test insecticide happens to be repellent. Instead, WHO susceptibility test kits were chosen as the exposure chamber for netting because use of these involves less mosquito handling, are easier to do and enable more precise exposures. For ease of handling the test netting was taped to a backing paper (same size as a WHO insecticide paper) before being inserted into the exposure tube and constrained with two metal rings. The kits' mesh lids were replaced with two layers of test netting to further reduce the ratio of untreated to treated surface inside the tube. Batches of 10 females were placed in the recovery tube of the test kit (which had been lined with plain paper). The mosquitoes were then blown into the exposure tube, kept there for three minutes, blown back into the recovery tube, and then held for 24 h together with a cotton wool pad soaked in sugar solution. Mortality was scored after 24 h. Tests were carried out using sugar-fed females of *Anopheles gambiae *Kisumu (insecticide susceptible), 2–5 days of age.

Seven replicates were carried out on each piece of netting, giving a total of 21 replicates (210 mosquitoes) for each treatment/wash combination.

### Chemical analysis

Netting squares measuring 5 cm × 5 cm were cut after the washing cycles had been completed. Deltamethrin was extracted using acetonitrile and the extract was injected (20 μl per sample of net) onto HPLC. The analyses were carried out using a Dionex Summit range of equipment and software (Camberly, Surrey, UK). Samples were separated on an Acclaim^R ^C_18 _120Å column (250 × 4.6 mm), eluted with water/acetonitrile (90:10%; v/v) at a flow rate of 2 ml/minutes and passed through a photodiode array detector (PDA-100) set at 275 nm. The authenticity of the detected peaks was determined by comparison of retention time, spectral extraction at 275 nm and spiking the sample with commercially available standard of deltamethrin. A calibration curve of deltamethrin was generated by Chromeleon (Dionex software) using known amounts of the standard (0–0.4 μg/ml) in acetonitrile injected onto the column. From this curve the amount of deltamethrin on the 25 cm^2 ^pieces was estimated and the dosage per m^2 ^calculated.

### Statistical analysis

The three-minute mortality tests were analysed using blocked logistic regression. The median time to knockdown tests and chemical assays were analysed using analysis of variance and *t*-tests. Bioassay and chemical assay results were compared using linear regression.

## Results

### Chemical analysis

**Figure 1:**
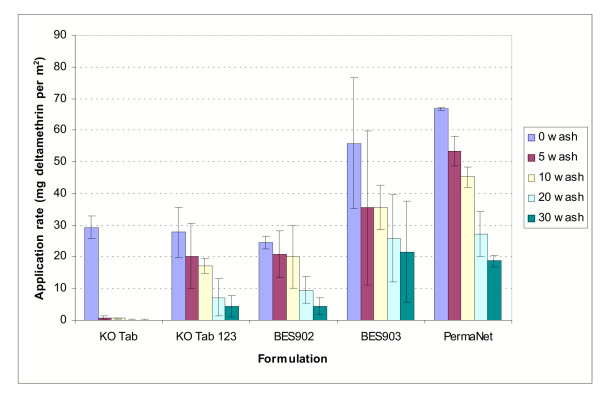
Mean (± CI) dosage of deltamethrin (mg/m^2^) before and after washing as measured using high-pressure liquid chromatography.

The results of HPLC analyses are presented in Figure 1. The loading dosage of deltamethrin on the KO-Tab treated net was close to the target of 25 mg/m^2^. The loading dosages of deltamethrin on the KO-Tab 1-2-3 treated net and on BES902 were within the same range of 25 mg/m^2^. The loading dosage on net BES903 was twice this amount (55.9 mg/m^2^), as this treatment was designed to resemble a PermaNet loading dosage. PermaNet itself showed a dosage of 66.7 mg/m^2^, somewhat higher than the manufacturer's target dosage of 55 mg/m^2^.

Washing mostly stripped the deltamethrin from the KO-Tab treated net. After 5 washes only 2.7% of the initial load remained, and after 10 washes only 1.7% (0.5 mg/m^2^) was present. After further washing residues were reduced to <0.2 mg/m^2^. With the KO-Tab 1-2-3 treated net, 73% of the initial load was still remaining after 5 washes and 62% was remaining at 10 washes. Only after 10 washes was there a more marked decline, with 25.6% remaining at 20 washes and 16% remaining at 30 washes.

With the net treated with formulation BES902, there was 83% still remaining at five washes and 81% at 10 washes. After 20 washes 38.5% remained.

By doubling the loading dose on net BES903 there was, at each wash interval examined, a comparatively higher dosage of deltamethrin still remaining compared to the KO-Tab 1-2-3 treated net and to net BES902. However, it is not clear why, after 30 washes, there was four times rather than two times as much active ingredient still remaining on net BES903 than on the KO-Tab 1-2-3 net or on net BES902.

With PermaNet the percentage of deltamethrin remaining was 80% of loading dose at five washes, 68% at 10 washes, 41% at 20 washes and 28% at 30 washes. At each of these wash points there was, relative to loading dose, proportionately more insecticide remaining on PermaNet than on the KO-Tab 1-2-3 treated net. With net BES903, on the other hand, the proportion of loading dose remaining at each wash point showed no clear difference from PermaNet. A notable difference between net BES903 and PermaNet was the narrower confidence interval on the PermaNet at each wash interval. This presumably represented a more homogeneous distribution of deltamethrin on the factory impregnated net than on the hand treated net.

### Three-minute exposure bioassay tests

**Figure 2:**
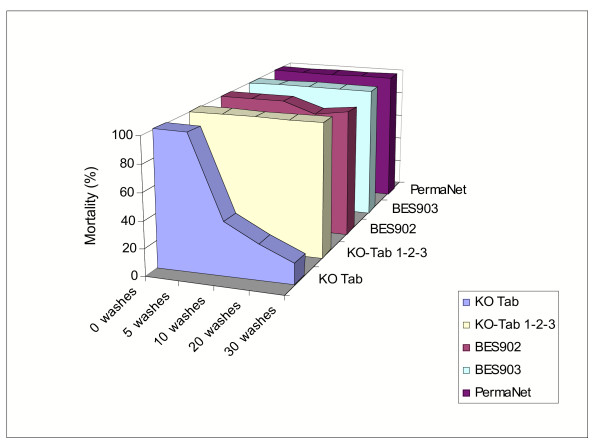
Three-minute exposure bioassay tests: proportion dead after 24 h. Seven replicate tests (70 mosquitoes exposed) were carried out on each formulation and wash interval.

Prior to washing the baseline mortality for all formulations was 100% (Figure 2). At 5 wash cycles mortality remained at 100%. At 10 wash cycles mortality on the KO-Tab treated net decreased to 39.4% and at 30 washes it decreased to 16.1%. On the nets treated with the long-lasting formulations (KO-Tab 1-2-3, BES902, BES903) 100% or near 100% mortality was observed after 30 wash cycles. PermaNet showed 100% mortality after 30 wash cycles.

HPLC analyses indicate that over 97% of deltamethrin was removed from the KO-Tab treated net after five washes (0.8 mg/m^2 ^remaining), whereas according to three-minute bioassay tests there was still sufficient active ingredient remaining to kill 100% after five washes.

### Median time to knock down tests

The results of median time to knockdown tests are shown in Figure 2. The time to knockdown of the median mosquito was quite consistent between replicates, as shown by narrow confidence intervals around the means of the medians. This enables small differences between treatments or washes to be detected using this technique.

The time to knockdown was fastest on unwashed nets, being inversely related to the amount of bioavailable deltamethrin. On unwashed netting the MTKD ranged from 461s on conventional KO-Tab to 502s on PermaNet and 560s on KO-Tab 1-2-3. Over the course of 30 washes the MTKD of conventional KO-Tab increased 2.4 fold to 1089s, whereas that of KO-Tab 1-2-3 only increased 1.3 fold to 742s and that of PermaNet only increased 1.1 fold to 554s. The increase in MTKD over 30 washes was significant for each of the treatments except for PermaNet which was borderline. For conventional KO-Tab the difference in MTKD over 30 washes (mean ± CI), 628 ± 99s (*P *< 0.0001), was greater than that of the long-lasting treatments; for KO-Tab 1-2-3 the difference was 181 ± 71s (*P *= 0.0001), for BES903 the difference was 136 ± 88s (*P *= 0.005) and for Permanet the difference was 52 ± 55s (*P *= 0.065). The difference in MTKD over 30 washes was therefore significantly less for PermaNet than for KO-Tab 1-2-3 or other Bayer long-lasting formulations.

**Figure 4:**
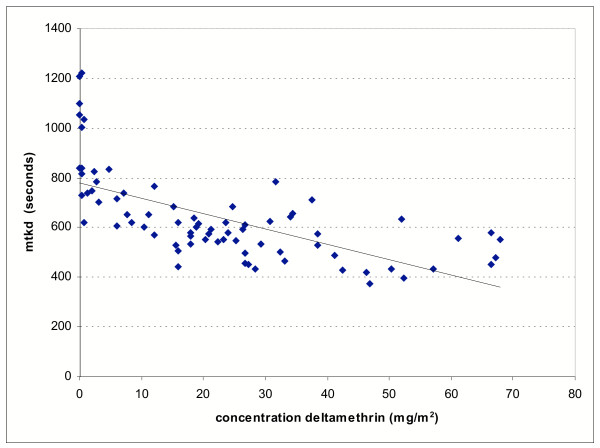
The relationship between median time to knockdown and dosage of deltamethrin.

**Figure 5:**
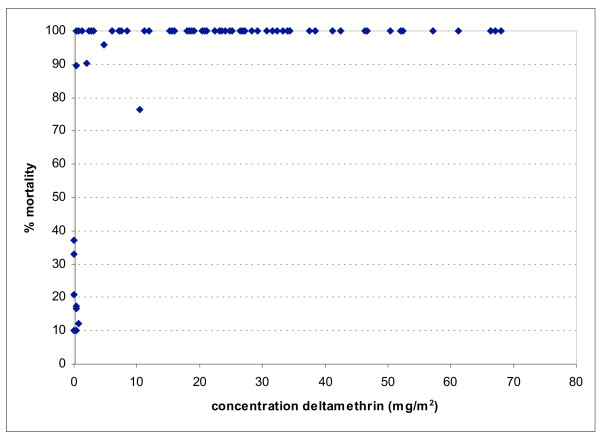
The relationship between % mortality (from three minutes bioassays) and dosage of deltamethrin.

Figure 4 shows that the MTKD test data are strongly correlated with the amount of deltamethrin detected on the nets using HPLC (P < 0.0001). The three-minute exposure bioassay results were also significantly correlated with deltamethrin content (P < 0.0001) but the relationship was less clear (Figure 5) owing to the consistently high mortalities observed with long lasting formulations over the 0–30 range of washes.

## Discussion

A long-lasting insecticidal net is defined by WHO as a net which gives greater than 80% mortality in three-minute exposure bioassays after 20 standardized washes [10]. Strictly speaking this should be after three minutes exposure in WHO cones rather than WHO cylinders. Cones were originally developed for assessing indoor residual spraying, in which 30 minutes exposure bioassays are conducted on firm flat surfaces such as sprayed walls [11]. For testing pyrethroid treated netting, cones have two disadvantages: pyrethroids are repellent and there is a large surface area within the cone that is non-insecticidal so exposure may be shorter than the intended three minutes; secondly, because insertion and removal of mosquitoes involves use of an aspirator it is harder to expose all mosquitoes for a precise three minutes period within the cone. For this reason some research groups are switching from cones to 'ball/wire frames' [11] or WHO cylinders as these techniques have neither of these disadvantages. WHO is currently sponsoring multicentre trials to assess the comparability of cones and cylinders for net bioassays. The work presented here pre-dates the WHO initiated trials. Comparing our results with cylinders with earlier studies with cones, it would seem that mortality in cylinders is indeed higher than in cones: for example, after subjecting PermaNet 2.0 to 20 standardized washes, Graham et al. [7] observed 81.8% mortality and Gonzales et al.[9] observed 87.1% mortality in cones whereas we observed 100% mortality in cylinders. More important are that tests are controlled, and the candidate LLIN is compared with a standard LLIN both of which are subjected to the same standardized washing procedures. Our evidence using cylinder tests is that KO-Tab 1-2-3 is a long-lasting wash-resistant treatment on a par with PermaNet in terms of mortality over 30 washes. But until we have a calibration curve for cylinders versus cones it cannot be said that a KO-Tab 1-2-3 treated polyester net is a long-lasting insecticidal net by the strict WHO definition.

Whereas the three minutes exposure bioassays indicated that KO-Tab 1-2-3 retains an insecticidal efficacy equivalent to PermaNet's over the course of 30 washes, HPLC analyses indicated that the wash resistance of KO-Tab 1-2-3 may decline at a faster rate between 10 and 30 washes. By contrast, PermaNet and BES903 were equivalent in wash fastness both in three minutes bioassay and chemical assay. It can be inferred that a one-day interval between washes was sufficient time for regeneration (if any regeneration was necessary) because there was sufficient insecticide available after each washing to still kill 100% of mosquitoes even though insecticide was being steadily removed. These are important findings that offer the hope of conventional ITN being made long lasting through simple dipping procedures that do not require sophisticated industrial processes or equipment.

The sensitive median time to knockdown bioassays supported the findings of the HPLC analyses and revealed incremental differences in insecticide performance after washing that three minutes exposure tests did not pick up. Comparing median time to knockdown over the course of 0-30 washes, KO-Tab 1-2-3 showed an additional 129 second increase in MTKD over PermaNet. At this stage of evaluation it is not clear whether the increased MTKD or greater loss of active ingredient in KO Tab 1-2-3 over 30 washes would translate to a poorer performance compared to PermaNet under field conditions.

The biological efficacy of the conventional KO-Tab treated net continued long after most of the insecticide had been removed by washing. The apparent discrepancy between biological and chemical assay results has been observed before in other comparative trials of long-lasting nets and conventionally treated nets [7]. It would seem that a small amount of bioavailable pyrethroid remains quite firmly bound to the net after most of the insecticide has been washed off. This residue is clearly sufficient to kill all mosquitoes that come into tarsal contact with it. One way to understand how this could be is to consider that 'feet get equally wet whether one goes paddling in the sea or step in a puddle of rainwater'.

Two further challenges await KO-Tab 1-2-3. Can this formulation render other types of polymer or fabric long-lasting? Will the long lasting treatment stand up to wear and tear and routine washing over the lifetime of a net in normal everyday use? There is great diversity in the fabrics and materials used for making mosquito nets, particularly in traditional net using societies that were using nets before the ITN revolution of the last two decades. The utility and wash resistance of KO-Tab 1-2-3 needs to be confirmed on nets made of cotton, nylon, polyethylene and other synthetic materials before this product can have the widest possible application or impact against malaria. There might also be potential in treating non-net substrates such as curtains, canvas tents or blankets.

The next phase of testing of KO-Tab 1-2-3 should be an evaluation in experimental huts of treated polyester nets washed up to 20 times, as recommended in recent WHO guidelines[10]. The ultimate demonstration of the persistence and effectiveness of this product would be during normal household use over at least 3 years in a variety of cultural, environmental and epidemiological situations. In view of the time-scales required for evaluating LLIN, such studies should run concurrently with phase 2 experimental hut trials.

## Authors' contributions

AY prepared the netting samples at LSHTM, undertook the median time to knockdown and chemical assays, summarised the data. RN supervised the mortality bioassays in Benin. HK carried out the HPLC chemical analysis. MR designed the study, analysed the data and prepared the manuscript.

**Figure 3:**
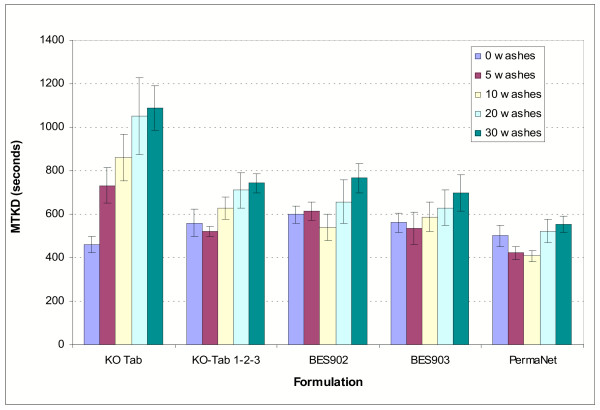
Median time to knockdown tests: means and confidence intervals. Nine replicate tests were carried out on each formulation and wash interval.
